# Supplementary material for: Predictive attenuation of touch and tactile gating are distinct perceptual phenomena
Source: iScience. 2022 Mar 14;25(4):104077. doi: 10.1016/j.isci.2022.104077 (PMC8968059; doi:10.1016/j.isci.2022.104077)
Supplement: Document S1. Figures S1–S7 and Text S1 [file mmc1.pdf]

iScience, Volume *25*

Supplemental information

Predictive attenuation of touch and tactile gating are distinct perceptual phenomena

Konstantina Kilteni and H. Henrik Ehrsson

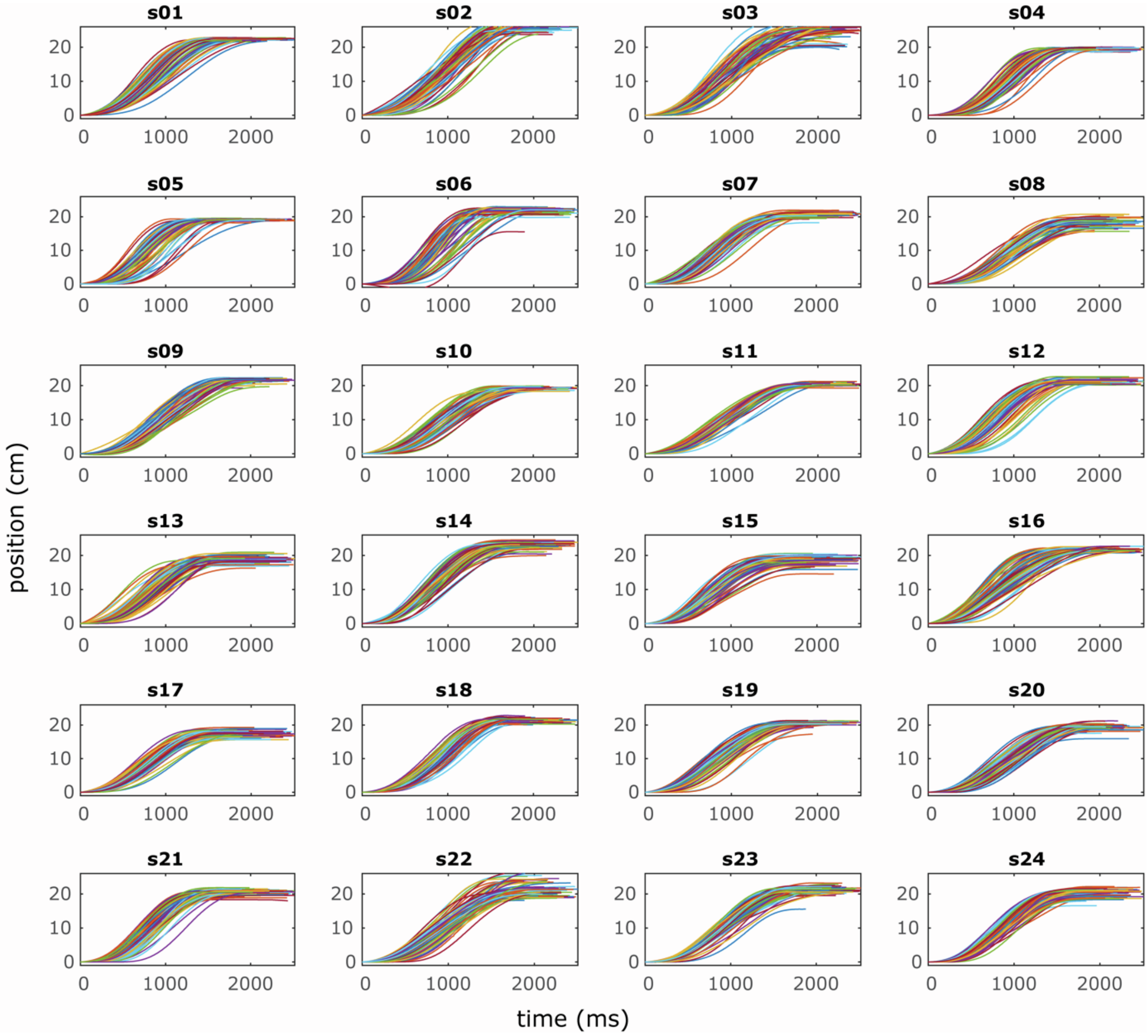

Fig. S1. Position traces for the participants' movements under the *gating* condition, related to Figure 1 and STAR Methods.

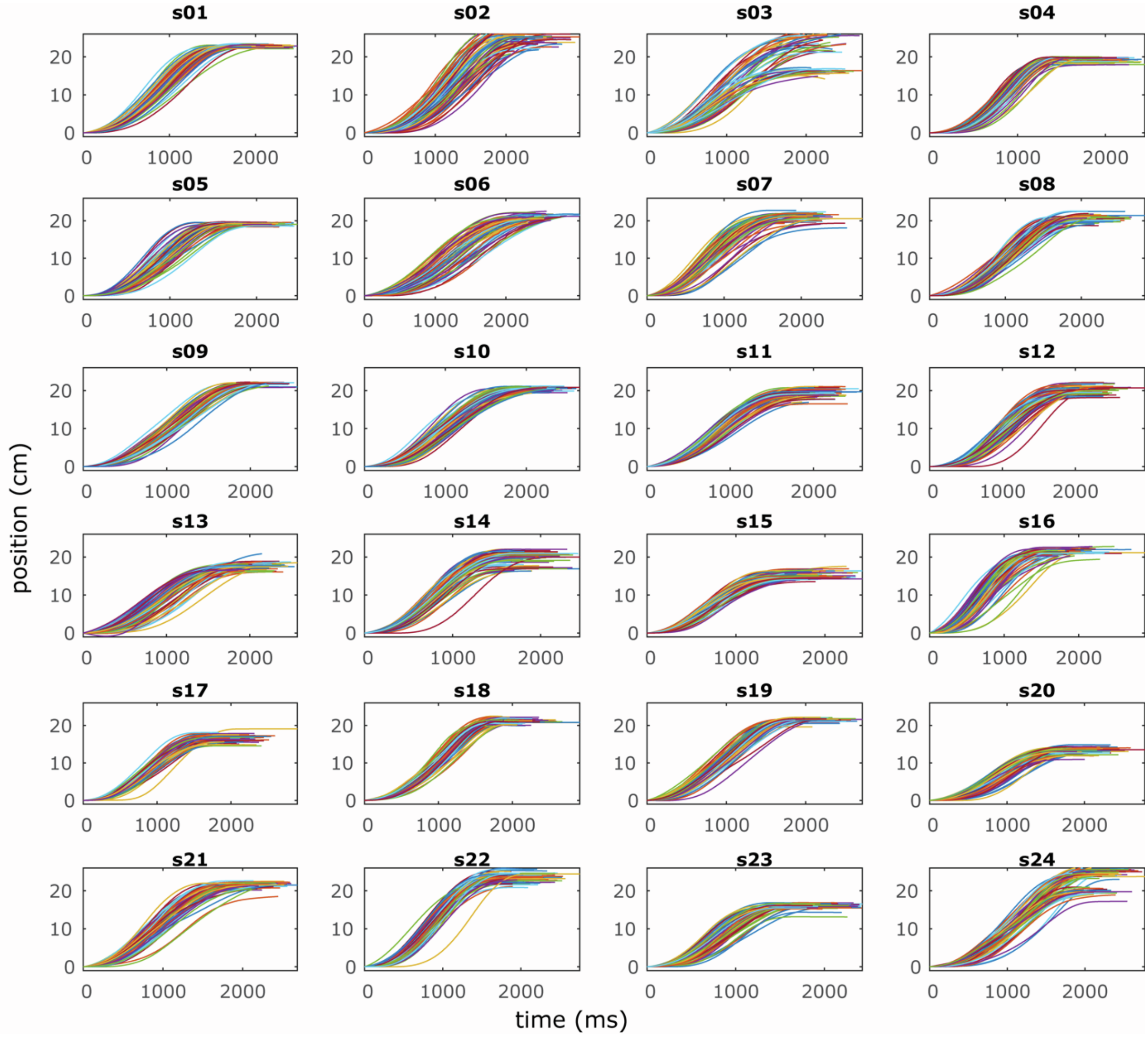

Fig. S2. Position traces for the participants' movements under the *attenuation&gating* condition, related to Figure 1 and STAR Methods.

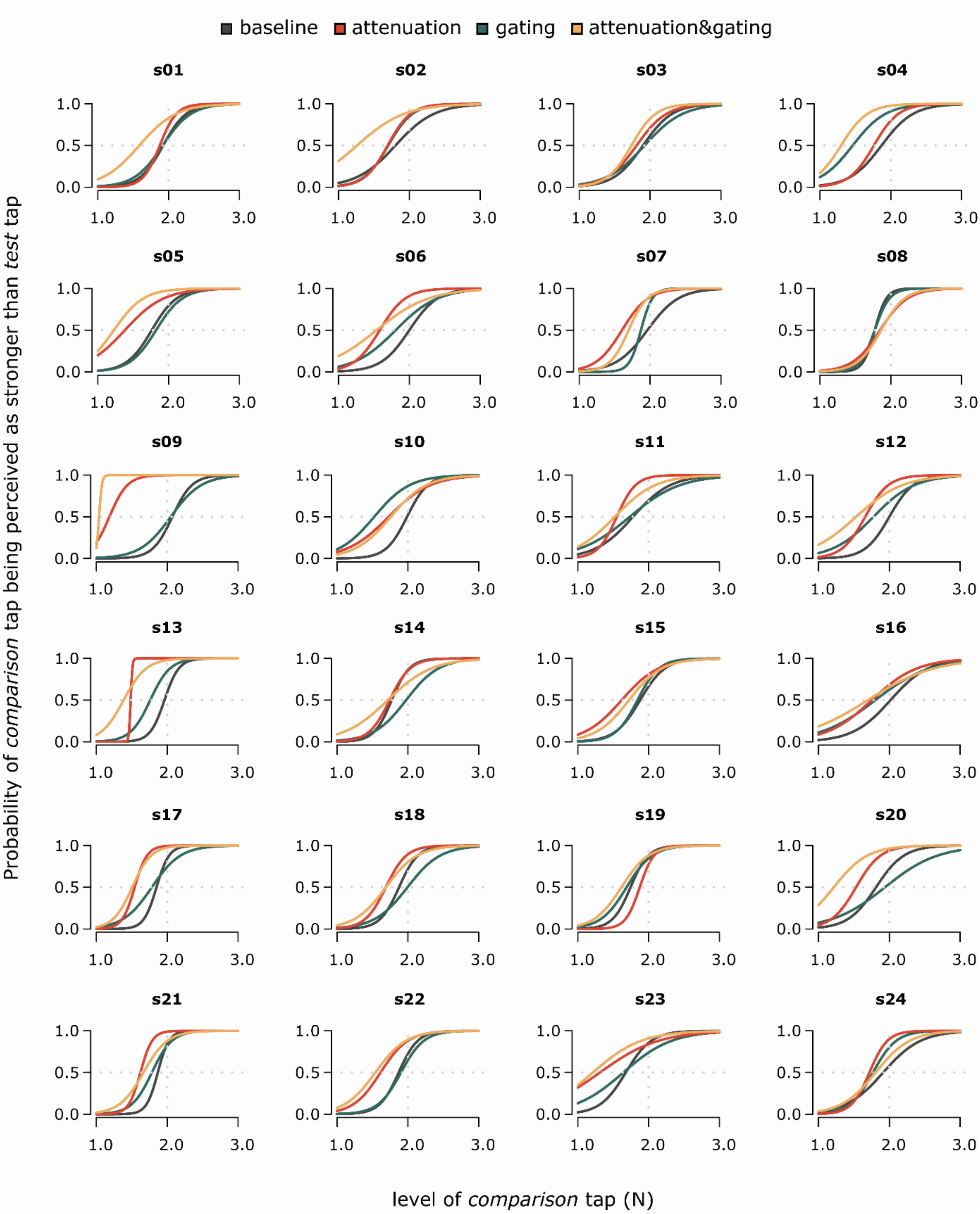

Fig. S3. Fitted logistic models based on the participants' responses under each condition, related to Figure 1, Figure 2 and STAR Methods.

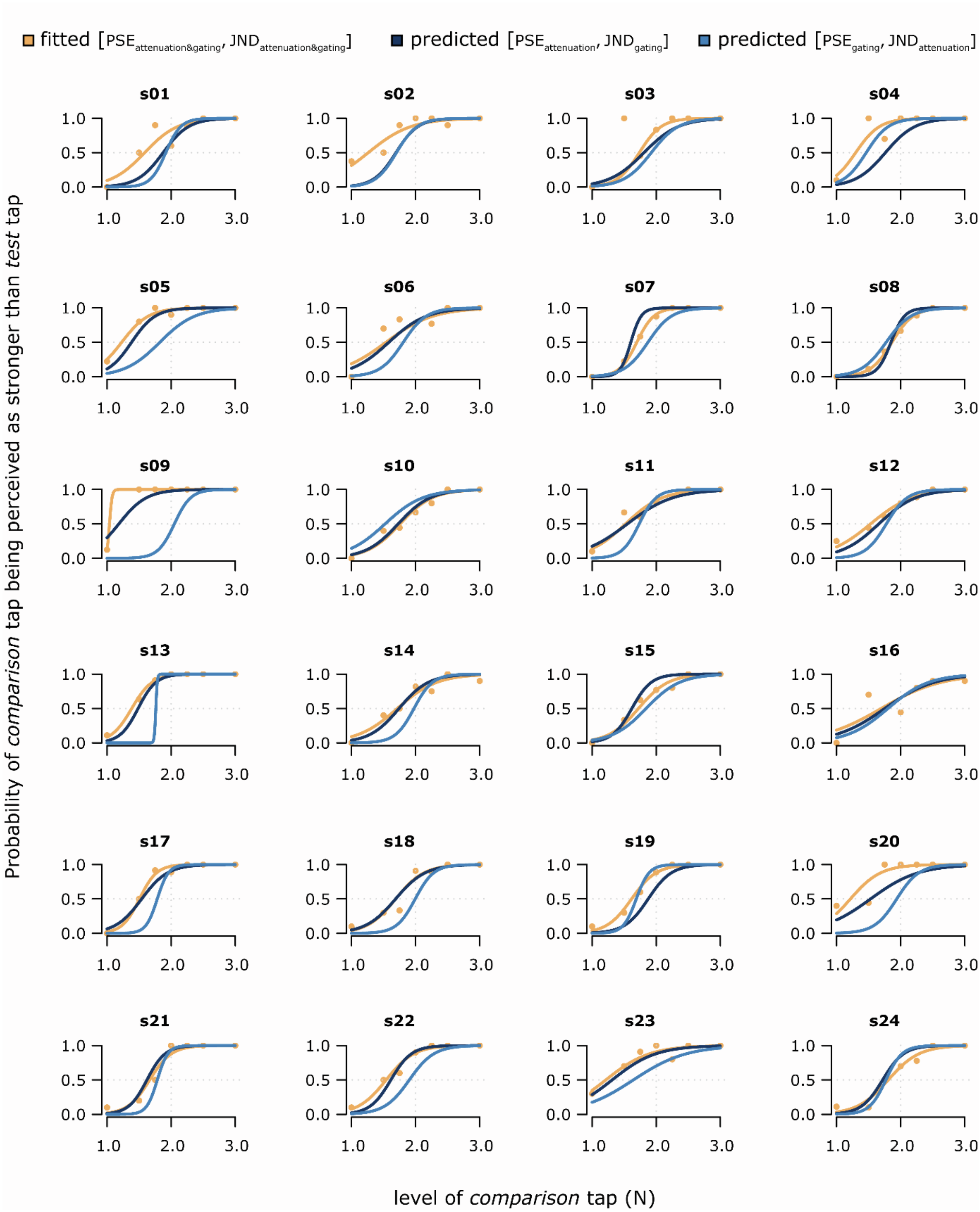

Fig. S4. Fitted logistic models based on the participants' responses under the *attenuation&gating* condition (yellow) and predicted logistic curves based on the participants' PSE and JND in the *attenuation* and *gating* conditions (blue), related to Figure 3.

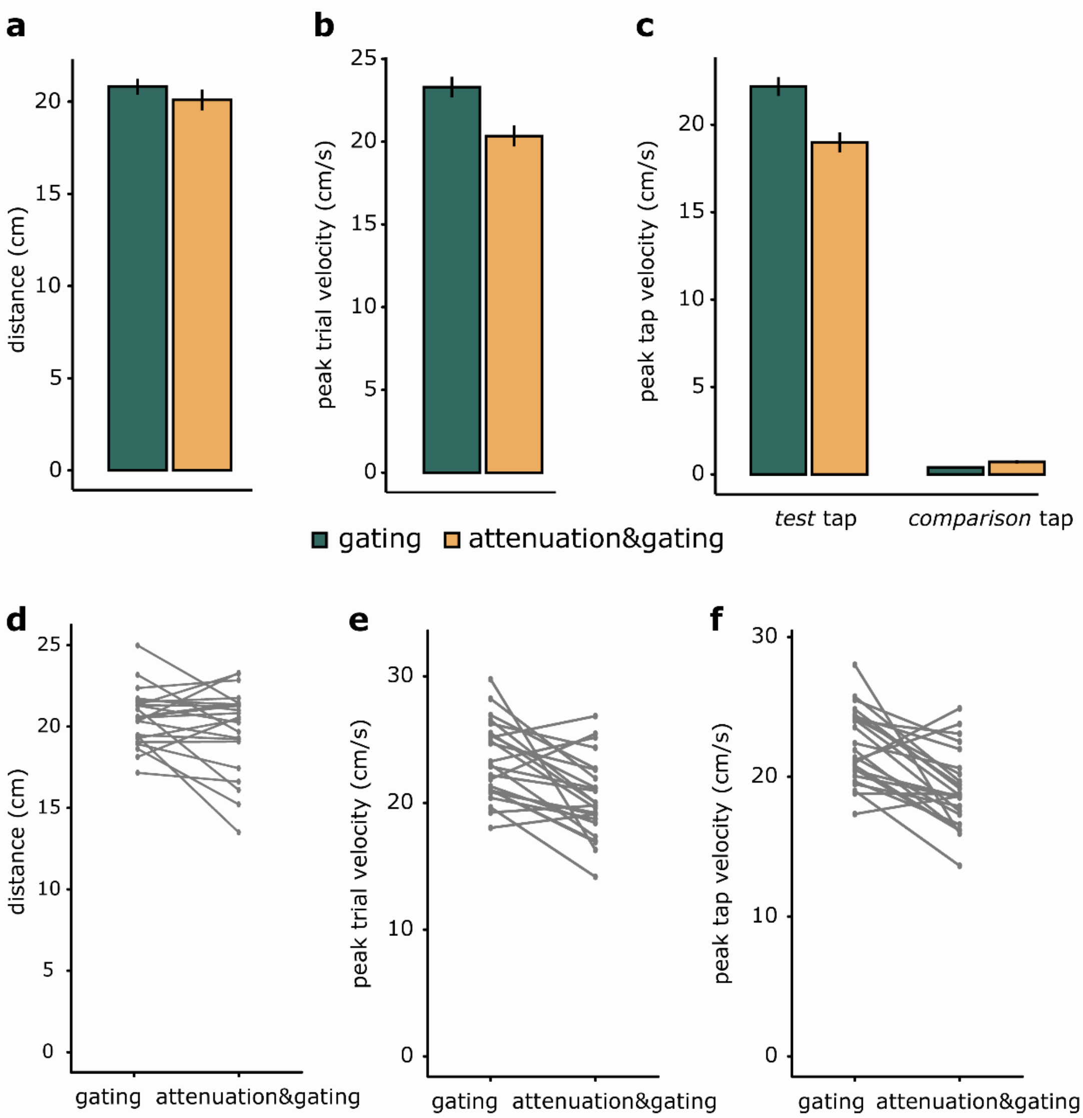

Fig. S5. Movement parameters for the *attenuation* and *attenuation&gating* conditions, related to STAR Methods. Bar graphs show **(a)** the total distance (mean ± SEM) run by the participants' left arm, **(b)** the peak velocity (mean ± SEM) for the entire trial duration, and **(c)** the peak velocity (mean ± SEM) at the intervals of the two taps. **(d, e, f)** Line plots illustrate the differences in distance, peak trial velocity and peak tap velocity between the *gating* and *attenuation&gating* conditions.

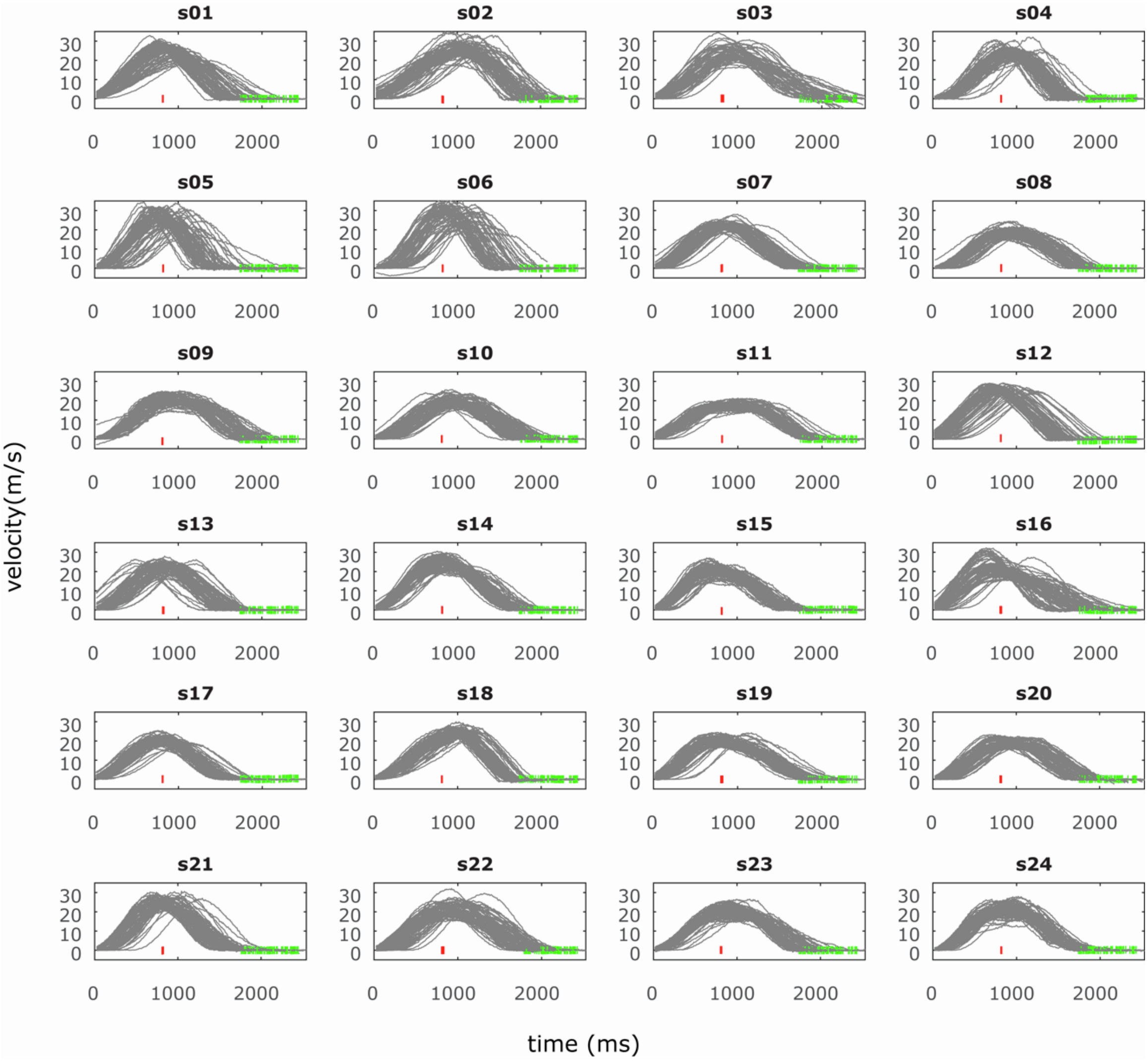

Fig. S6. Velocity profiles for the participants' movements under the *gating* condition, related to STAR Methods. Red and green lines indicate the times when the *test* tap and the *comparison* tap were delivered, respectively.

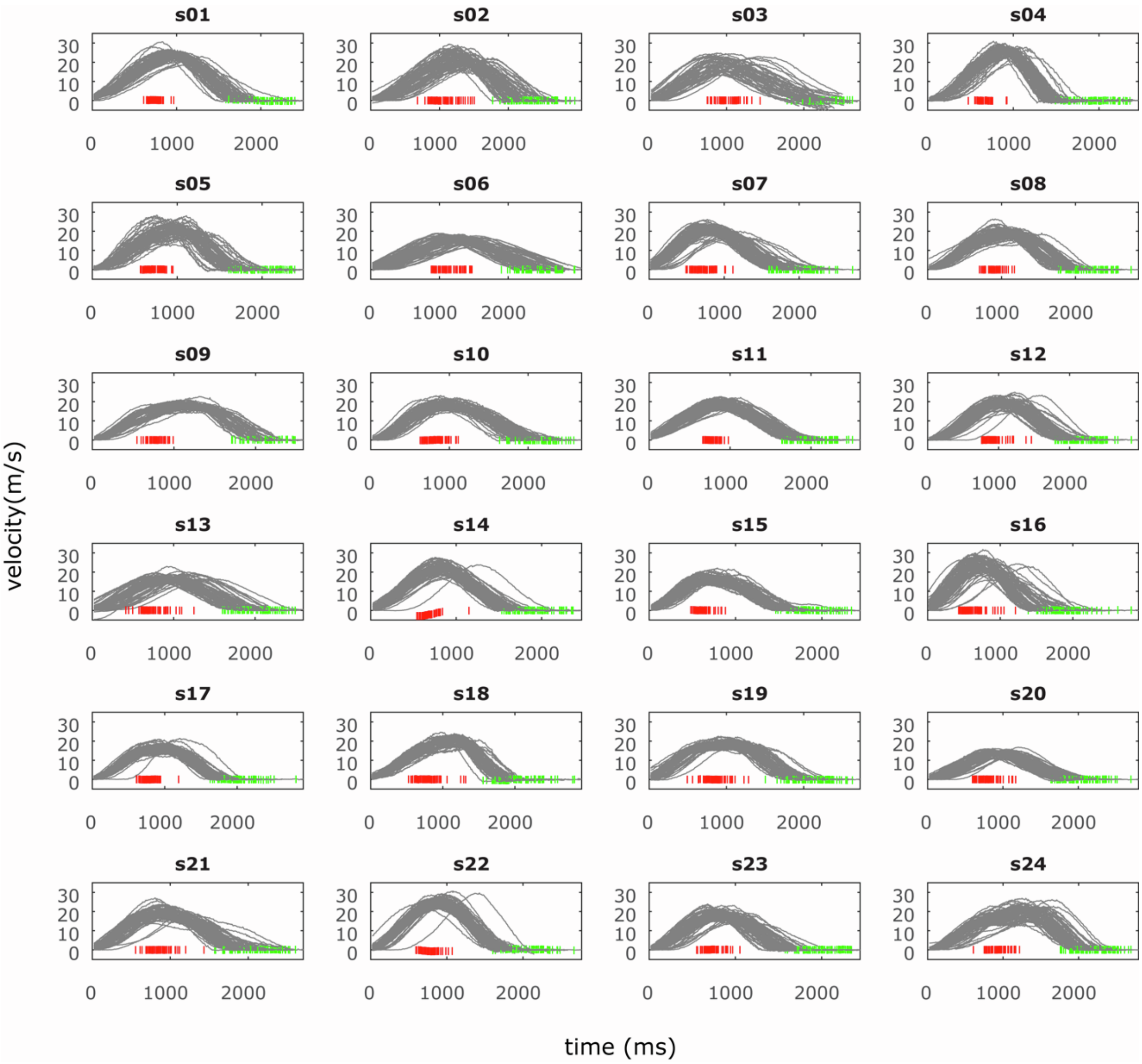

Fig. S7. Velocity profiles for the participants' movements under the *attenuation&gating* condition, related to STAR Methods. Red and green lines indicate the times when the *test* tap and the *comparison* tap were delivered, respectively.

Text S1. Consistency of effects

Although our planned analysis consisted of pairwise comparisons (see *Statistical analysis*), we also assessed the consistency of our PSE and JND effects by performing an exploratory post-hoc analysis using binomial tests. For this analysis, we assumed that the outcome of each participant is binary (success or failure). The null hypothesis indicates 50% success chance.

In the case of PSEs, we considered a decrease in PSE as 'success' and an increase (or no change) in PSE as 'failure'. Accordingly, 22 out of 24 participants (92%) decreased their PSE from the *baseline* to the *attenuation* condition (exact binomial test, $p < 0.001$), 18 out of 24 participants (75%) decreased their PSE from the *baseline* to the *gating* condition (exact binomial test, $p = 0.023$), and 23 out of 24 participants (96%) decreased their PSE from the *baseline* to the *attenuation&gating* condition (exact binomial test, $p < 0.001$). Moreover, 20 out of 24 participants (83%) decreased their PSE from the *gating* to the *attenuation* condition (exact binomial test, $p = 0.002$), 17 out of 24 participants (71%) decreased their PSE from the *attenuation* to the *attenuation&gating* condition (exact binomial test, $p = 0.064$), and 21 out of 24 participants (88%) decreased their PSE from the *gating* to the *attenuation&gating* condition (exact binomial test, $p < 0.001$).

In the case of JNDs, we considered an increase in JND as 'success' and a decrease (or no change) in JND as 'failure'. Accordingly, 11 out of 24 participants (46%) increased their JND from the *baseline* to the *attenuation* condition (exact binomial test, $p = 0.839$), 20 out of 24 participants (84%) increased their JND from the *baseline* to the *gating* condition (exact binomial test, $p = 0.002$), and 19 out of 24 participants (79%) increased their JND from the *baseline* to the *attenuation&gating* condition (exact binomial test, $p = 0.007$). Moreover, 17 out of 24 participants (71%) increased their JND from the *attenuation* to the *gating* condition (exact binomial test, $p = 0.064$), 16 out of 24 participants (67%) increased their JND from the *attenuation* to the *attenuation&gating* condition (exact binomial test, $p = 0.152$), and 16 out of 24 participants (67%) increased their JND from the *gating* to the *attenuation&gating* condition (exact binomial test, $p = 0.152$).
